# Antidepressants and Risk of Sudden Cardiac Death: A Network Meta-Analysis and Systematic Review

**DOI:** 10.3390/medsci9020026

**Published:** 2021-04-23

**Authors:** Narut Prasitlumkum, Wisit Cheungpasitporn, Nithi Tokavanich, Kimberly R. Ding, Jakrin Kewcharoen, Charat Thongprayoon, Wisit Kaewput, Tarun Bathini, Saraschandra Vallabhajosyula, Ronpichai Chokesuwattanaskul

**Affiliations:** 1 Division of Cardiology, University of California Riverside, Riverside, CA 92521, USA; narutprasitlumkum@gmail.com (N.P.); kding247@gmail.com (K.R.D.); 2 Department of Internal Medicine, Mayo Clinic, Rochester, MN 55902, USA; charat.thongprayoon@gmail.com; 3 Division of Cardiology, Department of Medicine, Faculty of Medicine, Chulalongkorn University and King Chulalongkorn Memorial Hospital, Thai Red Cross Society, Bangkok 10330, Thailand; tokavanich.n@gmail.com; 4 Department of Internal Medicine, University of Hawaii, Honolulu, HI 96822, USA; jakrinkewcharoen@gmail.com; 5 Department of Military and Community Medicine, Phramongkutklao College of Medicine, Bangkok 10400, Thailand; wisitnephro@gmail.com; 6 Department of Internal Medicine, University of Arizona, Tucson, AZ 85721, USA; tarunjacobb@gmail.com; 7 Section of Interventional Cardiology, Division of Cardiovascular Medicine, Department of Medicine, Emory University School of Medicine, Atlanta, GA 30322, USA; saraschandra.vallabhajosyula@emory.edu

**Keywords:** antidepressant, sudden cardiac death, network meta-analysis

## Abstract

*Background*: Antidepressants are one of the most prescribed medications, particularly for patients with mental disorders. Nevertheless, there are still limited data regarding the risk of ventricular arrhythmia (VA) and sudden cardiac death (SCD) associated with these medications. Thus, we performed systemic review and meta-analysis to characterize the risks of VA and SCD among patients who used common antidepressants. *Methods:* A literature search for studies that reported risk of ventricular arrhythmias and sudden cardiac death in antidepressant use from MEDLINE, EMBASE, and Cochrane Database from inception through September 2020. A random-effects model network meta-analysis model was used to analyze the relation between antidepressants and VA/SCD. Surface Under Cumulative Ranking Curve (SUCRA) was used to rank the treatment for each outcome. *Results:* The mean study sample size was 355,158 subjects. Tricyclic antidepressant (TCA) patients were the least likely to develop ventricular arrhythmia events/sudden cardiac deaths at OR 0.24, 0.028–1.2, OR 0.32 (95% CI 0.038–1.6) for serotonin and norepinephrine reuptake inhibitors (SNRI), and OR 0.36 (95% CI 0.043, 1.8) for selective serotonin reuptake inhibitors (SSRI), respectively. According to SUCRA analysis, TCA was on a higher rank compared to SNRI and SSRI considering the risk of VA/SCD. *Conclusion:* Our network meta-analysis demonstrated the low risk of VA/SCD among patients using antidepressants for SNRI, SSRI and especially, TCA. Despite the relatively lowest VA/SCD in TCA, drug efficacy and other adverse effects should be taken into account in patients with mental disorders.

## 1. Introduction

Owing to unclear pathophysiology in mood disorder, several neurochemical substances have been sought from time to time to explain this mythical entity, particularly roles of serotonergic, noradrenergic, dopaminergic, and cholinergic systems [1,2,3]. Moreover, not only an interplay between these neurohormonal systems, but also various genetic heterogeneities and expressions [4,5,6], are involved in this disease complexities. This has hence resulted in difficulties in establishing the most effective medical treatments for mood disorders. Nevertheless, it has been at least 50 years since the discovery of antidepressants and later led to several types of this particular group, despite only a 60% remission rate and delayed effective onset [7]. Common antidepressants including tricyclic antidepressants (TCAs), selective serotonin reuptake inhibitors (SSRIs), serotonin-norepinephrine reuptake inhibitors (SNRIs) and atypical groups have been widely used with acceptable safety profiles. However, use of these medications should be cautiously prescribed and monitored only by well-trained specialists as vital organs can be affected, for example, neuromuscular, respiratory and, importantly, cardiovascular systems. Antidepressants overdosage are also not uncommon, up to 3.2 per 10,000 persons with mortality rates 0.32% [8]. Moreover, paradoxical suicidal behavior from appropriate use has also been reported [9], emphasizing extra-careful surveillance and close follow-up.

Specifically, adverse cardiovascular effects of the medication have been well-documented since the 1980s and have been under continued surveillance. These include QT prolongation, Torsade de pointes (TdP) arrhythmias, Brugada syndrome phenotype, and polymorphic ventricular arrhythmias [10,11]. Independent literature studies have shown the risk of QT prolongation in *tricyclic antidepressants* (TCAs), *selective serotonin reuptake inhibitors* (SSRIs), *serotonin-norepinephrine reuptake inhibitors* (SNRIs), and various atypical antidepressants like mirtazapine and bupropion [12,13]. A QTc greater than 500 milliseconds has been associated with a two-fold increased risk for TdP, a contributory factor towards an increased length of hospital stay as well as mortality in patients [14]. Owing to scattered data related to ventricular arrhythmias and sudden cardiac death from antidepressants and a lack of head-to-head comparisons, we thus performed a network systematic review and meta-analysis to evaluate and analyze the overall likelihood of such risks collectively.

## 2. Methods

### 2.1. Literature Review and Search Strategy

A systematic literature search of MEDLINE (1946 to November 2020), EMBASE (1988 to November 2020), and the Cochrane Database of Systematic Reviews (database from inception to November 2020) was conducted to identify any relevant studies assessing the antidepressants and risks of SCD/VA.

The systematic literature review was undertaken independently by two investigators (R.C. and N.P.) applying a search approach that incorporated the terms of “ventricular arrhythmia” OR “sudden cardiac death” combined with the term “antidepressants” OR “SSRI” OR “TCA” OR “SNRI” which is provided in online Supplementary data A manual search for conceivably relevant studies using references of the included articles was also performed. No language limitation was applied. This study was conducted by the STROBE (Strengthening the Reporting of Observational Studies in Epidemiology) [15] and the Preferred Reporting Items for Systematic Reviews and Meta-Analysis (PRISMA) statement [16] (Table S2).

### 2.2. Selection Criteria

Eligible studies must be either observational studies (cohort, case-control, or cross-sectional studies) or randomized control trials that reported the risk of ventricular arrhythmia/sudden cardiac death and the use of antidepressants. They must provide data on the clinical characteristics, types of antidepressants, and events (either sudden cardiac death or ventricular arrhythmias). Inclusion was not limited by study size. Retrieved articles were individually reviewed for their eligibility by the two investigators (R.C. and N.P.). Discrepancies were discussed and resolved by a third researcher (N.T.). The Newcastle-Ottawa quality assessment scale was used to appraise the quality of study for case-control studies and outcomes of interest for cohort studies [17]. The modified Newcastle-Ottawa scale was used for cross-sectional studies [18]. The risk of bias by the Cochrane Collaboration’s tool [19] was used for assessing risk of bias for randomized trials as shown in Table 1.

### 2.3. Data Abstraction

A structured data collecting form was utilized to derive the following information from each study, including the title, the year of study, name of the first author, year of publication, the country where the study was conducted, demographic and characteristic data of patients using antidepressants, and incidence/prevalence of ventricular arrhythmias/sudden cardiac death.

### 2.4. Outcome of Interest

Our study was sought to assess the primary outcome which is the incidence of SCD/VA among patients who received any antidepressants.

### 2.5. Ancillary Information

For comprehensive review, we gathered and provided essential information inherent to acquired prolonged QT under the Table 2. Common causes and their countermeasures were further narrated.

### 2.6. Statistical Analysis

Analyses were performed using R software version 3.6.3 (R Foundation for Statistical Computing, Vienna, Austria). Adjusted point estimates from each included study were combined by the generic inverse variance approach of DerSimonian and Laird, which designated the weight of each study based on its variance [22]. According to the heterogeneous nature of included studies, for example different methods, inclusion criteria etc., we used a random-effects model rather than a fixed-effect model network meta-analysis model. To assess the magnitude of heterogeneity, we performed a comparison of the posterior distribution of the estimated heterogeneity variance with its predictive distribution. Surface Under Cumulative Ranking Curve (SUCRA) was used to rank the treatment for each outcome [23].

With respect to consistency evaluation (the agreement between direct and indirect evidence), we did a statistical evaluation by using a design by node splitting test. This consistency test allows us to confirm that the selection, or non-selection, of specific comparisons are not related to the true effect size of that comparison [24,25].

The Cochrane handbook of systematic review was used as reference for risk of bias assessment. Moreover, the Grading of Recommendations Assessment and the Development and Evaluation (GRADE) framework was performed to assess the certainty of information accounted for the network estimates of the main outcomes from individual studies [26].

We assessed if the primary outcome remained robust in a subgroup analysis by the sample size of individual study and study year [27]. The Brooks-Gelman_Rubin diagnostic was performed to assess the convergence of models.

A sensitivity analysis was performed by comparing the results between Frequentist Network Meta-Analysis approach and Bayesian Network Meta-Analysis approach.

## 3. Results

A total of 320 potentially eligible articles were identified using our search strategy. After the exclusion of 4 duplicated articles, case reports, correspondences, review articles, in vitro studies, pediatric patient population, and animal studies, 29 articles remained for full-length review. Twenty one were excluded from the full-length review as the outcomes of interest were not reported.

**Figure 1:**
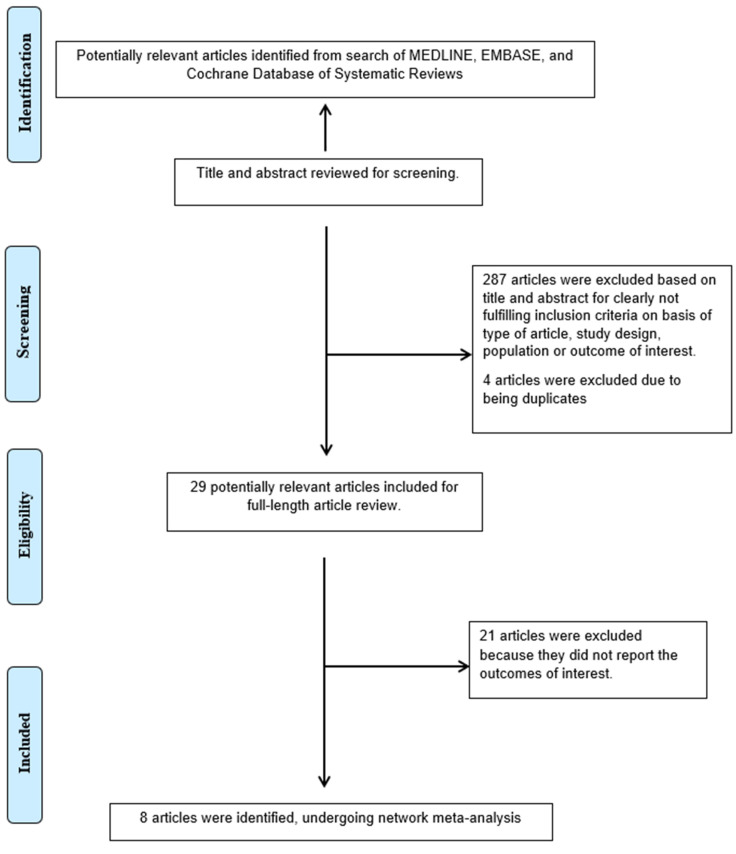
Outline of our search methodology.

Thus, the final analysis included in total eight studies (1 randomized study and 8 observational studies, including 5,682,348 patients. Five studies were conducted in Northern America while the rests were from Asia (Australia and Taiwan). The female population in our study ranged from 10–76%. The literature retrieval, review, and selection processes are demonstrated in Figure 1. The characteristics and quality assessment of the included studies are presented in Table 1, Table S1 and Figure S1.

**Figure 2:**
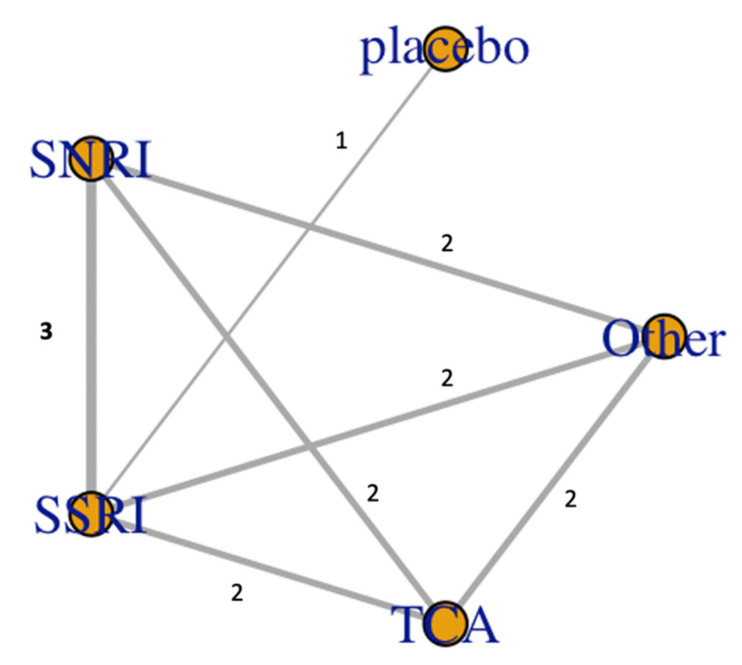
Network plot represented comparison among antidepressants, the network’s edge thickness corresponded with number of studies included (numbers on the edge are the number of included studies).

**Figure 3:**
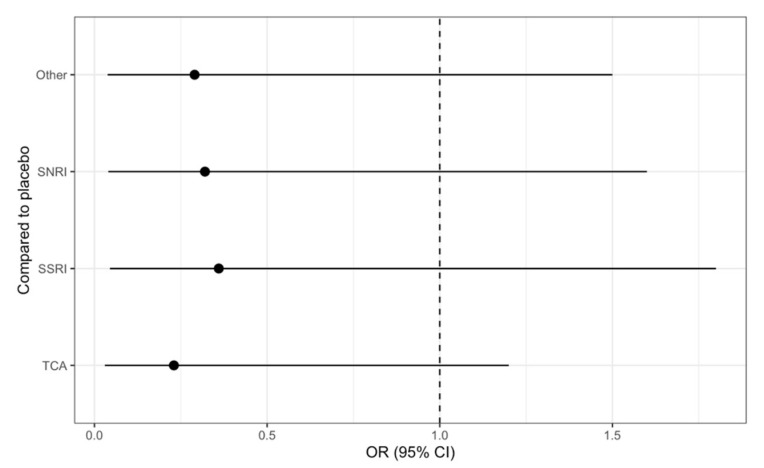
Forest plot demonstrated the relative effect of antidepressants compared to placebo.

**Figure 4:**
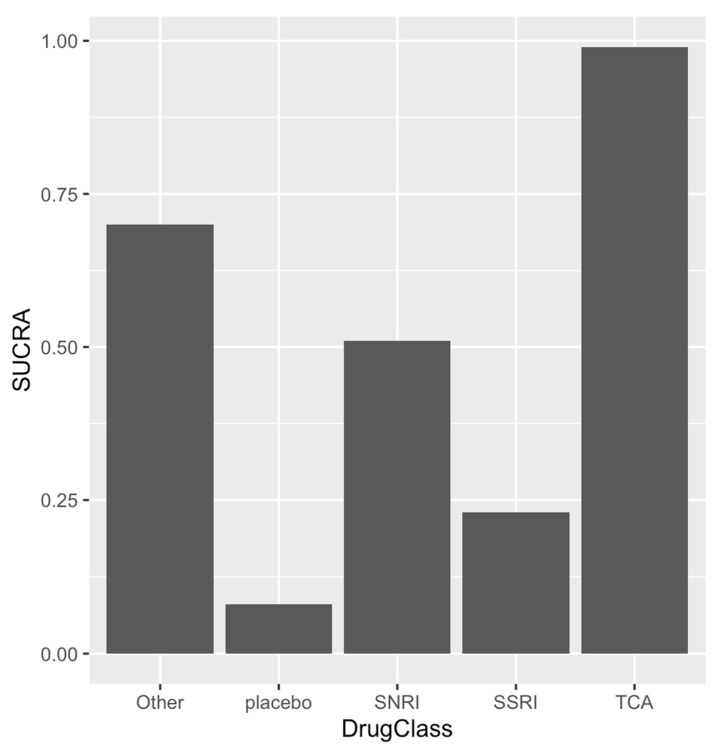
SUCRA graph rank the treatment options according to risk reduction in sudden cardiac death/ventricular arrhythmia.

The mean study sample size was 355,158 subjects. For individual antidepressants, 3,731,262 patients were assigned to SSRI, 371,726 for SNRI, 626,315 for the tricyclic antidepressant (TCA), and 187 for the placebo. The network plot, displayed in Figure 2, showed total number of studies and their head-to-head comparison Figure 3 forest plot demonstrated the relative effect of events compared to the placebo, TCA patients were least likely to develop ventricular arrhythmia events/sudden cardiac deaths at OR 0.24, 0.028–1.2, OR 0.32 (95% CI 0.038–1.6) for SNRI, and OR 0.36 (95% CI 0.043, 1.8) for SSRI, respectively. This was suggested with SUCRA graph, which demonstrated TCA was associated with VA/SCD risks (Figure 4).

The Q statistic was calculated to evaluate the consistency of the model, which revealed a non-significant *p* value (0.63). This supports consistency, which is the null hypothesis.

A sensitivity analysis was performed by comparing the result of NMA between the Bayesian method and the Frequentist method. Both statistical methods provided the same result, that TCA tends to have the least propensity to ventricular arrhythmia events/sudden cardiac death.

Testing for meta regression by excluding a large sample size study was performed. The result was still the same by demonstrating the relatively lower odds of ventricular arrhythmia events/sudden cardiac death in the TCA group compared to other antidepressants.

## 4. Discussion

In the current study, we demonstrated that the use of antidepressants in patients with mental disorders was not associated with VA/SCD. TCA has the lowest risk, followed by SNRI and SSRI in comparison with a placebo. We performed a sensitivity analysis as well as meta-regression, and the result was consistent with those before doing sensitivity analysis and meta-regression. The similar outcomes confirmed the low likelihood of VA/SCD from antidepressant use, especially TCA.

Primitively, TCA was the very first among antidepressant agents approved by Food and Drug Administration (FDA) for major depressive disorder in 1959 [7]. It has several pharmacological actions, inhibiting mainly through norepinephrine, serotonin reuptake receptors as well as interfering postsynaptic adrenergic alpha, muscarinic and histamine receptors [28]. Trading off with the efficacy in controlling mood disorder, undesirable effects are to be expected owing to its complex interactions. To the current date, there have been several antidepressant classes developed which have better side effect profiles compared to TCA, which had the highest dropout rates of up to 20% [29]. Nevertheless, TCA has at least equivalent, or even higher, performance compared to those other classes based on the largest network meta-analysis in 2018 [30]. Moreover, despite the TCA’s notoriety from its side effects and poor tolerance, another large meta-analysis with total participants of up to 380,000 showed no differences in all-cause mortality and CV mortality among major antidepressants classes, including SNRI, SSRI and TCA [31]. Moreover, one old study reported even superior efficacy in TCA compared to SSRI despite lower tolerability [32].

In fact, our study’s results also portrayed the same trend as previous studies, suggesting the lower risk of SCD/VA among patients using TCA [33,34,35]. Despite sounding counterintuitive since former studies showed high toxicity tendency amongst TCA use [36,37,38], these studies were based on overdose or high-dose usage. Practically, TCA was prescribed in dosages lower than the recommended (75 mg/day), mean doses of Amitriptyline (55.6 mg/day) and doxepin (47.8 mg/day) from the study [39]. In fact, TCA is not the first-line medication in mood disorder treatments but rather an adjunct when other medications fail to be effective [40,41]. Interestingly, the former study [42] showed lower adverse outcomes among TCA users in comparison with SSRI users.

There are a few reasons that may contribute to a relatively lower risk of VA in the TCA group. First, physicians cautiously use and closely monitor the side effects of TCA. They usually discontinue TCA as soon as EKG abnormalities hint at the toxicity of TCA. Second, the dose might be lower than we prescribed to achieve the same therapeutic target because multimodality approaches have been introduced to mitigate individual treatment doses. Third, when compared to TCA, SSRI and SNRI may not be highly selective enough for central serotonin receptors. Hence, this chemical interaction may dominantly occur peripherally, possibly ensuing CV intolerable effects such as bradycardia, QT prolongation and VA [43].

Fourth, SSRI has been putatively known as the most common antidepressant prescribed for mood disorders [44,45]. Nevertheless, several studies [46,47,48] demonstrated the lethal side effects from SSRI, particularly a prolonged QT interval and fatal arrhythmia. Moreover, some studies reported other cardiovascular adverse effects including Prinzmetal angina, orthostatic hypotension, and an intraventricular conduction defect [49,50]. It should be noted that, despite these known serious effects, SSRI is considered safe when dosages are within the therapeutic range [51]. Patients presented with fatal arrhythmia, TdP, usually had predisposing conditions such as electrolyte imbalance, coronary artery disease and polypharmacy issues [52]. Furthermore, recent meta-analyses emphasized SSRI’s tolerability and neutral effects on cardiovascular outcomes [53,54], reflecting their safety profile in clinical practice.

As an emerging antidepressant commonly used as a single-agent or in combination with other classes, SNRI has been not well studied yet, particularly focusing on its cardiac adverse outcomes in a susceptible population [31,55]. SNRI indirectly stimulates sympathetic pathways by limiting sympathetic neurotransmitter reuptake from the synaptic cleft. This response leads to rising blood pressure and tachycardia. Overstimulating may cause cardiovascular systems to have a hypertensive crisis/fatal arrhythmia [56]. However, side effects of SNRI have been rarely reported. Recent studies [46,57] portrayed safe cardiovascular effects from SNRI, with seemingly well-tolerated and lessened side effects.

Our study has several limitations. First, the studies included in our analysis were all observational studies with different demographics and methodologies, of which residual bias cannot be excluded. Nevertheless, this is the largest pool of data among patients with depression who were prescribed antidepressants. We also performed different network meta-analysis approaches—the Bayesian and Frequentist method— to minimize the bias. Our objective is to provide the most updated information regarding antidepressants’ safety. Second, the lack of variable adjustments may potentially result in over/underestimated information. Hence, we performed meta-regression and sensitivity analysis, which demonstrated consistent results. Third, the lack of ECG information to determine SCD/VA risk markers from each antidepressant precluded the explanation of links between medications and the risk of SVD/VA in this study, especially degrees of QR prolongation, the frequency of PVCs, and their differences among each medication classes. Fourth, this study was a class-effect analysis thus minor differences in agents among each class may ensue. Moreover, the introduction of newer antidepressants generations might portend clinical changes, particularly better acceptability, which is the main purpose for these newer agents. Regardless, one report by Parcher et al. [58] crucially pointed out the contrasting results from one would expect. Several case reports compiled in this study suggested putative unfavorable CV safety in these newer antidepressants’ generation especially prolonged QT related arrhythmia. Hence, we believe the unaltered result would be yielded if sufficient data are retrievable. Fifth, dosages of medications were not adjusted in our analysis owing to inhomogeneous reports as well as data insufficiency. This is unfortunately our major limitation and one should be cognizant interpreting this piece of evidence. Despite the matters, we believe our analysis reflected real-world practices where most physicians comfortably preferred the lowest but safest dosages to this population.

## 5. Conclusions

This is the first and largest network meta-analysis to demonstrate the comparatively low risk of VA/SCD among patients using antidepressant, for SNRI, SSRI and especially, TCA. Despite the notoriety of these medications, cautious use per recommendations would provide treatment benefits rather than toxicities.

## Figures and Tables

**Table 1:** Study Characteristics.

Author	Country	Type of Study	Sex (Female%)	Participants	Mean Age (Years)	Mean Follow Up (Months)	Medications Ascertainment	Outcome Ascertainment	NOS
Angerman 2016	USA	RCT	24	HF patients with depression	62	18.5	Data and safety committee	Chart review adjudicated by steering committee	Referred to supplementary
Leonerd 2011	USA	Prospective cohort study	N/A	Antidepressant users from Medicaid data from 5 large states	N/A	N/A	Mapping national drug code to Lexicom	ICD-9	8
Martinez 2010	Australia	Case-control	56.7	New antidepressant users from UK database	72.9	39.6	Prescription records from UK-GPRD	Read/OXMIS codes	9
Qirjazi 2016	Canada	Retrospective cohort study	66	Participants who took SSRI	76	3	Data from the Ontario Drug Benefit database	ICD-10	6
Ray 2017	USA	Retrospective cohort study	76	Participants who took high-dose SSRI	47	8.4	Tennessee Medicaid files	death certificate-Medicaid enrollment and ICD 9/10	8
Lin 2019	Taiwan	Retrospective cohort study	60	Participants with depression who took medications	N/A	N/A	Longitudinal Health Insurance Databases	ICD-9	7
Wu 2017	Taiwan	Retrospective cohort study	63.9	Participants with depression who took medications	N/A	2.5	Taiwan’s National Health Insurance Research Database	ICD-9CM	7
Zivin 2013	USA	Retrospective cohort study	9.6	VA Participants with depression who took medications	N/A	N/A	VHA National Registry for Depression	ICD-9	8

Abbreviations: CM: Clinical modifications; HF: Heart failure; ICD: International classifications of diseases; N/A: Not available; NOS: Newcastle-Ottawa scale; OXMIS: Oxford Medical Information Systems; RCT: Randomized control trial; SSRI: Serotonin selective reuptake inhibitor; UK: United Kingdom; UK-GPRD: United Kingdom General Practice Research Database; USA: United states of America; VHA: Veteran health administration.

**Table 2:** Acquired conditions predisposing QT prolongation and TdP [20,21].

Conditions		Countermeasures
Metabolic derangements	Hypokalemia, Hypomagnesemia, Hypocalcemia	Correct electrolytes
Bradyarrhythmias	Sick sinus syndrome, AV block	Correct reversible causes, Pacemaker implantation
Antidepressants	TCA, SSRI, SRNI, atypical antidepressants such as Trazodone, Atomoxetine	Avoidance, removal, serial ECG monitoring
Antipsychotics	Haloperidol, Clozapine, Chlorpromazine, Risperidone, thioridazine	Avoidance, removal, serial ECG monitoring,
Antiarrhythmic drugs	Class Ia, Ic and class III	Avoidance, removal, serial ECG monitoring,
Antibiotics	Macrolides, Fluroquinolones, Azoles, Quinines, Quinidine, Antiretroviral drugs	Avoidance, removal, serial ECG monitoring,
Gastrointestinal drugs	Cisapride, Metoclopramide, Domperidone, Ondansetron	Avoidance, removal, serial ECG monitoring,
Others	Myocardial infarction, increased intracranial pressures, hypothermia, organophosphate poisoning, cocaine intoxication,	Address underlying, Avoidance offending agents, antidotes

## Data Availability

The data presented in this study are available on request from the corresponding author.

## Supplementary Materials

The following are available online at https://www.mdpi.com/article/10.3390/medsci9020026/s1, Supplementary data: Search term, Table S1: Newcastle Ottawa quality assessment scales, Figure S1: Risk of bias graph for randomized control trial studies, Table S2: PRISMA checklist.

## Sample Availability

Samples of the compounds are not available from the authors.

## Abbreviations

ECG	Electrocardiogram
NMA	Network meta-analysis
QTc	Corrected QT duration
SCD	Sudden cardiac death
SNRI	Serotonin-norepinephrine reuptake inhibitors
SSRI	selective serotonin reuptake inhibitors
TCA	Tricyclic antidepressants
TdP	Torsade de Pointes
VA	Ventricular arrhythmias
